# Viral Assemblages of a Hypersaline Estuary Show Divergent Responses to Freshwater and Temperature Disturbances

**DOI:** 10.1111/1758-2229.70354

**Published:** 2026-05-08

**Authors:** Jordan R. Walker, Paxton T. Bachand, Jeffrey W. Turner, Jessica M. Labonté

**Affiliations:** ^1^ Department of Marine Biology Texas A&M University at Galveston Galveston Texas USA; ^2^ Department of Biology University of Miami Coral Gables Florida USA; ^3^ Department of Life Sciences Texas A&M University‐Corpus Christi Corpus Christi Texas USA

**Keywords:** disturbance, hypersaline, microbiome, salinity, viromes, viruses

## Abstract

Hypersaline environments harbor extremely dense bacterial and viral populations unique from other aquatic ecosystems. Changes to the hydrologic cycle and anthropogenic disturbances have the potential to alter these poorly described communities. Here, we aimed to assess the variation within the viral and bacterial communities of one of the world's largest hypersaline estuaries over 13 months. Using metagenomics, we identified viruses associated with two different salinity regimes, and we showed how viruses responded to pulse disturbances including freshwater inundation and freeze events. We identified 17, 324 viral species, of which 12,132 were found in only one of the salinity regimes. Our results demonstrate a potential association between freshwater pulses throughout June 2021 and shifts in viral community composition. Freeze events showed a greater propensity to alter the auxiliary metabolic genes (AMGs), or genes carried by viruses to alter host metabolism during infection. Viruses associated with low temperatures led to higher incidences of AMGs associated with sulfur cycling and oxidative phosphorylation as opposed to photosynthesis with freshwater inundation and no extreme weather. The contrasting responses to different pulse disturbances make evident the need to better understand how different types of disturbances alter viral communities and their potential to modulate important biogeochemical cycles.

## Graphical abstract

Metagenomic assessment of viromes at two sites in Laguna Madre, a hypersaline estuary, showed unique viral communities. Extreme cold temperature changes showed a greater propensity to enrich AMGs toward oxidative phosphorylation and sulfur metabolism. Extreme shifts in salinity led to homogenized viral taxonomic groups more similar to those typically found at the less saline site and higher incidence of photosynthesis AMGs.

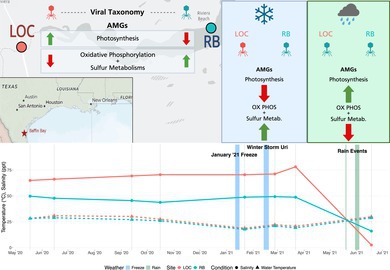

## 1 Introduction

Hypersaline environments can attain high microbial densities, reaching 10^12^ cells per mL, despite their extreme salinities (Brum et al. 2005; Guixa‐Boixareu et al. 1996; Paul and Mormile 2017; Pedrós‐Alió et al. 2000; Williams 2002). As with many aquatic ecosystems, viruses in hypersaline environments tend to outnumber microbial cells by one to two orders of magnitude, and estimations of the densest viral communities range from 10^9^ to 10^12^ viruses per mL (Brum et al. 2005; Guixa‐Boixareu et al. 1996). Despite such high concentrations, viral infection rates in hypersaline systems are low (Brum et al. 2005). The low viral infection rates and low protozoan grazing may explain the high microbial abundances of these systems (Brum et al. 2005; Pedrós‐Alió et al. 2000). Hypotheses to explain the high viral density but low viral infectivity paradigm within these systems include larger burst sizes (Guixa‐Boixareu et al. 1996), high viral diversity leading to lower contact rates with hosts (Brum et al. 2005), higher levels of phage defence systems (Brum et al. 2005; Moller and Liang 2017), and higher viral survival rates due to increased glycerol production by phytoplankton common in hypersaline environments (Bettarel et al. 2011).

Hypersaline environments are threatened by a changing hydrological cycle due to anthropogenic interference (Paul and Mormile 2017; Williams 2002). The predicted increased frequency of droughts with a warming climate exacerbated by increased water demand and decreased freshwater inflows threatens to alter historically hypersaline environments by making them more saline or decreasing their area (Paul and Mormile 2017; Williams 2002). Viral morphology and community composition have been shown to be linked to salinity increases within hypersaline communities (Bettarel et al. 2011; Emerson et al. 2012; Roux et al. 2016). Viral communities from different geographies share similar gene content; however, when comparing viruses at the nucleotide level, they are found to be unique across regional scales (Dávila‐Ramos et al. 2019; Emerson et al. 2012; Roux et al. 2016). Therefore, the indirect effect of salinity on hosts and their associated viruses selects for similar gene content and functions despite globally distributed hypersaline environments sharing few viral species or strains (Roux et al. 2016). Despite the current evidence that salinity can drive viral community composition, less is known about how seasonal changes and pulse disturbances may alter viral communities in hypersaline environments.

Global climate change brings the threat of increased rates of extreme rainfall and flooding, resulting in more frequent and extreme shifts in salinity within these hypersaline systems (Coumou and Rahmstorf 2012; Donat et al. 2016; Knutson et al. 2015). The magnitude of a freshwater pulse can determine how the microbial communities respond to the disturbance, with high magnitude inflows favoring fast‐growing constituents and smaller pulses favoring metabolically diverse microorganisms that respond to nutrient input quickly (Dorado et al. 2015; Paerl et al. 2020; Roelke et al. 2013). Extreme rainfall has been shown to alter the taxonomic composition of microbial communities, while functional redundancy maintains ecosystem function (Uritskiy et al. 2019; Walker et al. 2022). Furthermore, dilution events can alter viral communities through prophage induction (Motlagh et al. 2017; Santos et al. 2011), introduction of transient viruses, decreasing infection rates, and temporarily altering viral functional potential (Williamson et al. 2014; Woods et al. 2022). However, there is a need to better understand the impact of freshwater inundation on hypersaline communities, given the threat climate change poses to these unique environments.

Here, we aimed to characterize the viral community composition in one of the world's largest hypersaline estuaries. The Laguna Madre, located near Corpus Christi, Texas, United States, is a shallow, sub‐tropical system and the only estuary in North America where salinities regularly exceed that of seawater (Cuddy and Dunton 2023). Despite their importance, hypersaline coastal bays remain poorly characterized with respect to viral community structure. The Laguna Madre is known to experience a myriad of disturbance events including droughts, floods, freeze events, and heatwaves. The timing of the sampling allowed to evaluate the potential impacts of disturbance events on viral communities. We sampled two sites over 13 months: Los Olmos Creek (LOC), a shallow tributary, where salinities fluctuated between 2–82 ppt (mean = 60 ppt) and Riviera Beach (RB), where salinities fluctuated between 16 and 50 ppt (mean = 42 ppt). We specifically aimed to determine if the viral community composition and function were similar between sites, how the viral communities varied between samplings, and if extreme weather events would alter the composition of the viral communities. This pilot dataset provides the first snapshot of viral diversity in Baffin Bay and establishes a baseline against which future seasonal and spatial studies can be compared.

## 2 Materials and Methods

### 2.1 Sampling Collection

**FIGURE 1:**
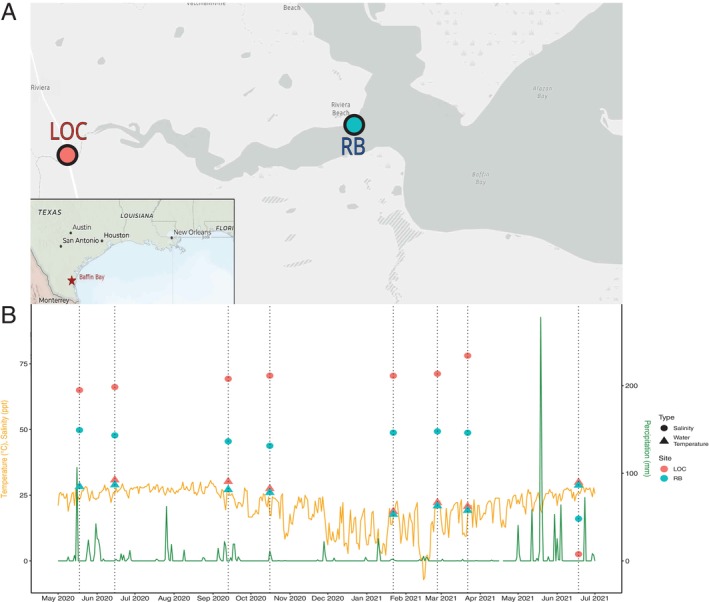
(A) Map of the sampling sites at Los Olmos Creek (LOC) in red and Riviera Beach (RB) in blue. (B) Line graph of the temperature (orange) and precipitation (green) measurements from NOAA's Kingsville station. Dotted lines indicate sampling dates. Circles indicate salinity measured and triangles air temperature measured at the time of sampling at LOC (red).

Water samples were collected from the Los Olmos Creek (LOC) site (27°16′23.62″ N, 97°48′08.01″ W) and the Riviera Beach (RB) site (27°17′00.09″ N, 97°39′52.80″ W) in the Baffin Bay watershed (Figure 1A). The LOC site is a shallow, typically hypersaline tributary that flows into the Laguna Salada (Tunnell and Judd 2002) and a well‐documented reservoir for persistent brown tide blooms (Cira and Wetz 2019). The RB site is a public recreational area at the Laguna Salada and Baffin Bay confluence, 13.7 km from the LOC site. Sampling was conducted over 13 months, from May 18, 2020, to June 18, 2021 (Figure 1B, Table 1). Sampling efforts were impaired by a global pandemic. Sampling campaigns were therefore intermittent, and eight samples were chosen as representative from each season (Figure 1B). Daily air temperature and precipitation data were collected from NOAA's Climate Data Online portal from the Kingsville, TX station which is the closest NOAA observation station within 25 miles (Figure 1B).

**TABLE 1:** Abiotic conditions associated with each site.

Month	Site	Season	Temp (°C)	DO (mg/L)	Salinity (ppt)	pH	NH_4_ (μM)	NO_3_ (μM)	NO_2_ (μM)	Auto‐fluorescing cells/mL
May 20	LOC	Spring	28.33	5.86	64.99	8.3	44.35 ± 4.25	2.4 ± 0.14	0 ± 0	80,000
June 20	LOC	Summer	30.8	4.12	66.16	8.55	47.77 ± 2.76	2.1 ± 0.05	0 ± 0	60,000
Sept 20	LOC	Fall	30.2	0.7	69.3	8.12	36.9 ± 4.03	1.8 ± 0.14	0 ± 0	85,000
Oct 20	LOC	Fall	27.5	0.57	70.5	7.9	8.21 ± 0.26	3.11 ± 0.32	0.08 ± 0.05	35,000
Jan 21	LOC	Winter	18.8	3.25	70.46	8.33	14.09 ± 0.91	4.55 ± 0.19	0.82 ± 0.02	225,000
Feb 21	LOC	Winter	22.2	4.45	71.22	8.38	9.61 ± 1.03	9.04 ± 0.4	0.05 ± 0.01	2,225,000
Mar 21	LOC	Spring	20.7	4.74	78.1	7.65	6.68 ± 0.99	10.68 ± 0.19	0.05 ± 0.03	1,025,000
June 21	LOC	Summer	29.9	2.33	2.57	8.66	8.7 ± 0.93	2.19 ± 0.57	0.05 ± 0.01	25,000
May 20	RB	Spring	28.24	7.8	49.78	8.32	180.67 ± 0	2.83 ± 0.21	0.01 ± 0.01	35,000
June 20	RB	Summer	28.93	6.53	47.77	8.17	156.48 ± 35.87	2.45 ± 0.21	0.06 ± 0	5000
Sept 20	RB	Fall	27.1	4.41	45.5	7.91	161.38 ± 31.97	2.05 ± 0.04	0 ± 0	10,000
Oct 20	RB	Fall	26	4.12	43.8	8.06	22.37 ± 0.35	2.4 ± 0.64	0.27 ± 0.09	5000
Jan 21	RB	Winter	17.7	6	48.8	8.12	21.46 ± 1.86	3.9 ± 0.18	0.11 ± 0.01	200,000
Feb 21	RB	Winter	20.93	7.25	49.3	8.17	17.49 ± 0.49	7.45 ± 0.26	0.19 ± 0.24	15,000
Mar 21	RB	Spring	19.2	7.15	48.77	8.02	19.16 ± 0.36	13.03 ± 0.31	0.09 ± 0.09	15,000
June 21	RB	Summer	28.8	5.92	16.05	8.37	30.39 ± 0.11	1.81 ± 0.12	0.02 ± 0.02	20,000

### 2.2 Water Quality Parameters

Water temperature (°C), pH, dissolved oxygen (%), and salinity were recorded with a YSI 556 Multi Probe System (YSI Incorporated, Yellow Springs, OH, United States). For identification and quantification of different nitrogen species, water samples were filtered through a 0.22 μm PES filter and concentrations of NO_3_^−^, NO_2_^−^, and NH_4_^+^ were measured using a SEAL AQ300 Discrete Analyser (AQ300 methods EPA‐148‐D, EPA‐115‐D, and EPA‐126‐D, respectively). Water samples (50 mL) were preserved with Lugol's solution (1% final concentration, v/v), and auto‐fluorescing cell abundances were estimated by directly counting five random cells of the center field with a Leica DM750 fluorescence microscope (Wetzlar, Germany) using a 0.1 mL hemacytometer at 400× magnification (Hall et al. 2018).

### 2.3 DNA Isolation

For the characterization of the viral communities, a single sample of approximately 2 L of water was pre‐filtered using Nitex filters (30 μm) and stored in 24 L acid‐washed, field‐rinsed carboys. Due to the high biomass in the LOC samples, each sample was first pre‐filtered through 142 mm GF/D or GF/F (0.7 or 2.7 μm) to remove larger particles. The filtrate was then filtered through 142 mm PVDF (0.22 μm) to remove bacterial cells. The final filtrate, which contained viral particles, was concentrated using the iron flocculation method (John et al. 2011; Poulos et al. 2018). Briefly, the filtrate was treated with 100 μL of 10 g/L iron chloride stock solution, vigorously shaken, and incubated for 1 h. The samples were then filtered through 142 mm 0.8 μm hydrophilic polyethersulfone filters and stored at 4°C. Filters were treated with an oxalic acid buffer (0.15 M Tris‐base, 0.1 M Na_2_ ‐EDTA, 0.2 M MgCl_2_ hexahydrate, 0.2 M oxalic acid, pH = 6) and stored overnight at 4°C. The buffer was passed through a 0.2 μm syringe filter and stored at 4°C until DNA extraction. Viruses were further concentrated to a final volume of < 1 mL using 30 kDa molecular weight cutoff 50 mL centrifugal filtration units (Amicon Ultra‐15, Millipore). The Wizard Plus Miniprep DNA Purification System (Promega, Madison, WI, United States) was used to extract DNA from the virus concentrates using an adapted protocol (Poulos et al. 2018). DNA extracts were purified using magnetic beads (Sera‐mag SpeedBeads, Cytiva, Marlborough, MA, United States) (Faircloth and Glenn 2011) before being stored at −20°C.

For the characterization of the microbial communities, a single water sample (100 mL) was filtered through low‐protein binding 0.22 μm polyethersulfone (PES) filters (MilliporeSigma, Burlington, MA, United States). Total genomic DNA was isolated from the filters using a DNeasy PowerSoil kit (QIAGEN, Hilden, Germany) according to the manufacturer's instructions. The DNA quantity (ng/μL) and quality (A260/A280 and A260/A230 absorbance ratios) were measured using a biospectrophotometer (Bio‐Rad, Hercules, CA, United States), and the DNA was stored at −20°C until sequencing.

### 2.4 Metagenomic Sequencing and Analysis

DNA (~444 ng per sample) from the eight viral concentrates (viral fraction) and four 0.22 μm PES filters (microbial fraction) was sequenced at the Texas A&M Genomics and Bioinformatics facility using Illumina NovaSeq 6000 SP (150 bp paired‐ends) technology. Quality control of the raw sequencing files, which consisted of predicting sequence adapters, merging reads, trimming sequencing adapters and artifacts, removing low quality reads, masking low complexity regions, and removing common laboratory contaminants, was performed using BBTools (Bushnell et al. 2017). Reads were assembled using the de novo sequence assembler MEGAHIT using both the merged and unmerged quality‐controlled sequences (Li et al. 2015). Quality control and assembly statistics are shown in Table S1. Viral sequences were predicted from both fractions utilizing VIBRANT (Kieft et al. 2020), Virorter2 (Guo et al. 2021), CheckV (Nayfach, Camargo, et al. 2021), and geNomad (Camargo, Roux, et al. 2023). Results of CheckV and Virsorter2 were filtered for contigs containing at least one viral sequence as identified by CheckV. The results of the four programs were concatenated and deduplicated using Dedupe in BBTools. Some viral prediction softwares trim contigs and create duplicate sequences that Dedupe does not recognize; therefore, we screened contigs based on contig name and retained the shortest sequence. The resulting viruses were filtered to keep contigs > 10 kbp and sequences identified in at least two of the viral prediction programs. While the decision to retain only viral contigs > 10 kb likely removes the ssDNA viruses and leads to lower sensitivity in the detection of viral populations, it significantly reduces false discovery and over‐estimation of viral population richness (Campillo‐Balderas et al. 2015; Roux et al. 2019, 2017). Viral contigs were then clustered at 95% ANI and 85% alignment coverage to create viral operational taxonomic units (vOTUs) (Nayfach, Páez‐Espino, et al. 2021). Reads were mapped on the contigs using BWA‐MEM. For vOTUs with > 75% coverage in a sample, the number of reads mapped was calculated with samtools (Danecek et al. 2021; Vasimuddin et al. 2019). Taxonomy of the vOTUs was predicted with diamond blastp to align the sequences against the complete IMGVR version 7 database using the “more sensitive” setting (Buchfink et al. 2021; Camargo, Nayfach, et al. 2023). Taxonomic ranks were assigned to each vOTU if more than 50% of the annotated genes on a contig came from a particular taxonomic rank; those that did not were categorized as “*unknown*” of the lowest predicted taxonomic level. To predict and annotate auxiliary metabolic genes (AMGs), Virsorter2 was used with the following options ‐seqname‐suffix‐off viral‐gene‐enrich‐off, provirus‐off, prep‐for, and min‐score equal to 0.5 against all the available databases, followed by the DRAM‐v annotate and distill pipelines (Shaffer et al. 2020).

Metagenome assembled genomes were created by mapping the quality reads within the bacterial fractions on the contigs. We then used Metabat2, Metadecoder, and SemiBin to generate MAGs (Kang et al. 2019; Liu et al. 2022; Pan et al. 2022). The contigs within each MAG were then filtered to remove sequences identified as viral in the previous steps to avoid false positive virus–host prediction. The results of this analysis were then used in DASTool to refine the MAGs (Sieber et al. 2018). The results of DASTool contained no MAGs with < 50% completion, and MAGs with > 10% contamination, as predicted by DASTool. The quality MAGs (medium = > 50% completion, <10% contamination and high = > 90% completion, < 5% contamination) were analyzed with Anvi'o‐8 metagenomics workflow (Eren et al. 2021). MAG taxonomy was predicted by identifying and annotating single copy genes in Anvi'o and CAT/BAT (von Meijenfeldt et al. 2019). Results of both analyses were compared for consistency. While there was no disagreement between the two analyses, some predictions were at lower taxonomic levels, and the highest taxonomic ranks were retained. Finally, virus–host predictions were made using iPHoP with the default settings, and the August 2023 database was amended with MAGs from DASTool (Roux et al. 2023). The results of iPHoP were filtered to only retain virus–host matches between MAGs and vOTUs.

### 2.5 Statistical Analyses

MAGs and vOTU read counts were normalized to the length (kbp) of the MAG or viral contig and to millions of reads within each respective metagenome, from now on referred to as relative abundance. Venn diagrams and stacked bar histograms of the vOTUs and MAGs were constructed using ggplot2 and ggvenn in R (Team R. C 2024; Wickham 2016; Yan 2023). Diversity, richness, and evenness indices were calculated using vegan and fossil in R (Oksanen et al. 2024; Vavrek 2011). NMDS plots of the vOTUs, MAGs, AMGs, and abiotic conditions were constructed and analyzed using analysis of similarity, procrustes, and protest functions in vegan. The best fit model of abiotic conditions that predict the (dis)similarity of the MAGs and vOTUs was predicted using the bioenv() function in vegan. All correlation analyses used Pearson's correlation in the corrr package in R (Kuhn et al. 2024). PERMANOVA analysis was done to determine if samples significantly grouped by season or site using the adonis2 function in vegan. Clustering of the vOTUs based on read count data was conducted using the pipeline outlined in Coenen et al. (2020). Briefly, read counts, including those with < 75% coverage, were normalized, detrended, and scaled utilizing the DeSeq package, and then vOTUs were clustered using a k‐medoids approach (Love et al. 2014). K‐medoid dynamics and the relative abundance of vOTUs, viral families, and AMGs were visualized with ggplot2 and ComplexHeatmap (Gu 2022).

## 3 Results

### 3.1 Abiotic Conditions

A comparison of the abiotic conditions at each site revealed significant differences (α < 0.05; Wilcoxon Signed Ranks) in the mean values observed for water temperature, dissolved oxygen, salinity, transparency, ammonium, and auto‐fluorescing cells counted. The LOC site was on average 1.4°C warmer, 17.9 ppt more saline, and contained ~12.3× the auto‐fluorescing cells compared to the RB site (Table 1). At the RB site, dissolved oxygen percent saturation was on average 44 points higher, and ammonium levels were 3.5× higher than at the LOC site (Table 1). Two large freeze events occurred approximately one week prior to the January 2021 and February 2021 samplings; despite water temperatures remaining above 0°C, widespread fish and animal deaths due to low temperatures were observed. Following these samplings and continuing into March 2021, we observed significant increases in nitrate and nitrite and significantly lower levels of ammonium (Figure 1, Table 1). The June 2021 samples, following two consecutive large rain events, were the only samples where the salinity of either location fell below the threshold to be considered a hypersaline environment (32 ppt) (Figure 1, Table 1).

### 3.2 Sequencing Results

Each of the eight viral metagenomes or viromes generated ~40 M reads, assembled into 12.5 M contigs (total of 7.93 Gbp) with an average N50 of 726 bp (Table S1). In comparison, the four bacterial metagenomes generated ~45 M reads assembled into ~7.56 M contigs (total of 5.46 Gbp) and an average N50 of 853 bp (Table S1). Quality control measures including removal of sequencing artefacts, adapter trimming, and common laboratory contamination removal were consistent across samples (Table S1). Per sample assembly statistics were lower for the viral fraction than the bacterial fraction due to the generally small nature of viral genomes (Table S1).

### 3.3 Viral Community Composition

**FIGURE 2:**
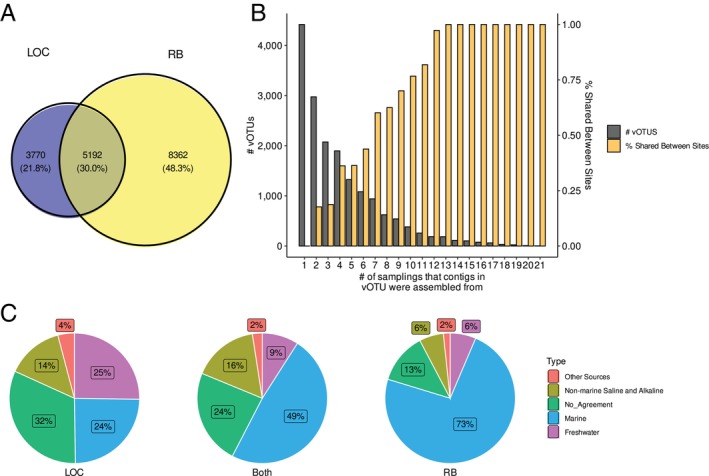
(A) Venn diagram of the number of vOTUs that contained contigs strictly from the LOC or RB sites or contained contigs from both. (B) Modified upset plot where the *x*‐axis represents the number of different metagenomes from which contigs within a vOTU were found. The left *y*‐axis represents the total number of vOTUs, and the right *y*‐axis represents the percentage of vOTUs that were found in both the LOC and RB sites. (C) Source environment of vOTUs determined by taxonomic assignment using IMGVR and the associated metadata.

We identified a total of 80,707 viral contigs which clustered into 17,324 vOTUs from both the viral and bacterial fractions. We found that 48% and 22% of the vOTUs were exclusively found at the RB and LOC sites, respectively (Figure 2A). Additionally, 25% of the vOTUs constructed only contained contigs from a single metagenome (Figure 2B). As the number of metagenomes represented in a vOTU increased, so did the likelihood that the vOTU had contigs generated from both sites (Figure 2B). RB vOTUs were dominated by viruses previously identified in marine samples. LOC vOTUs were more similar to viruses from freshwater and non‐marine saline and alkaline environments, or we were unable to reliably determine the origin (Figure 2C).

**FIGURE 3:**
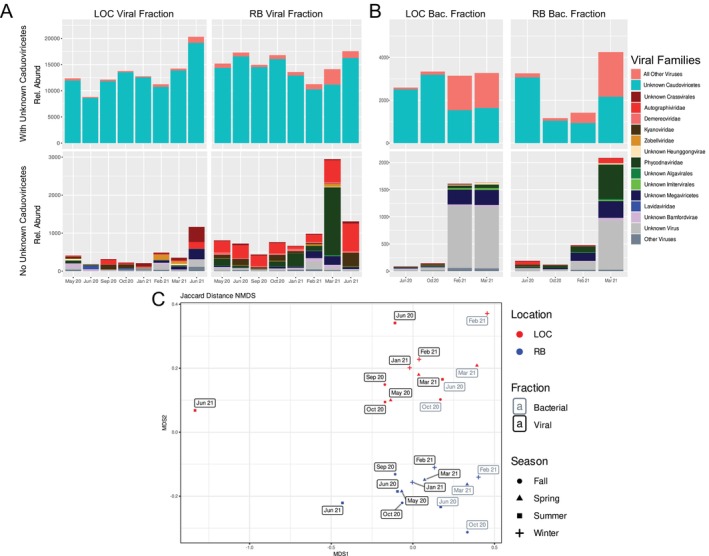
Histogram of the viral taxonomic composition of (A) the viromes and (B) the bacterial fractions where the top shows the amount of Unknown Caudoviricetes compared to any other viral family and the bottom shows the composition of the viral families that are not Unknown Caudoviricetes. (C) NMDS analysis of the centered log‐ratio transformed vOTU relative abundances using Euclidean distances (stress = 0.15).

Taxonomic classification of the vOTUs revealed that a majority of the vOTUs could only be classified down to the class level and were from Caudoviricetes (Figure 3A,B). The only samples with less than 90% of sequences from Caudoviricetes, unidentifiable at a lower taxonomic level, were viruses identified in bacterial samples from February and March 2021 and the March 2021 RB virome which were the two samples following the largest freeze event from February 13–17, 2021 (Figure 3B). vOTUs associated with a specific viral family, such as *Phycodnaviridae*, *Autographivirdae*, and *Kyanoviridae*, were more abundant at the RB site. There were more *Zobellviridae* and *Lavidaviradae* at the LOC site (Figure 3A,B). Diversity, evenness, and richness indices based on the vOTU relative abundances showed that the RB site contained a more diverse viral community compared to the LOC site, confirmed by paired Wilcoxon signed‐rank tests (α < 0.05) (Figure S1). NMDS analysis of the vOTUs relative abundances confirmed that the samples largely grouped according to the sampling site with a weaker trend in the temporal sampling and a clear separation of the samples collected post‐freshwater inundation in June 2021 (Figure 3C). Visual trends were confirmed with ANOSIM analysis, where grouping by seasons resulted in an R value 0.0897 (α = 0.08), while grouping by location resulted in an R value of 0.4734 (*α* = 0.0002). Comparison of the contextual conditions and the NMDS analysis of vOTU relative abundances showed significant correlations (α < 0.05) with salinity (*r* ^2^ = 0.52), temperature (*r* ^2^ = 0.61), nitrate (*r* ^2^ = 0.66), ammonium (*r* ^2^ = 0.40), and dissolved oxygen (*r* ^2^ = 0.56). To confirm these findings, we estimated the subset of variables that correlates maximally with the (dis)‐similarity and found that salinity had the maximum correlation with a Spearman rank correlation of 0.70.

**FIGURE 4:**
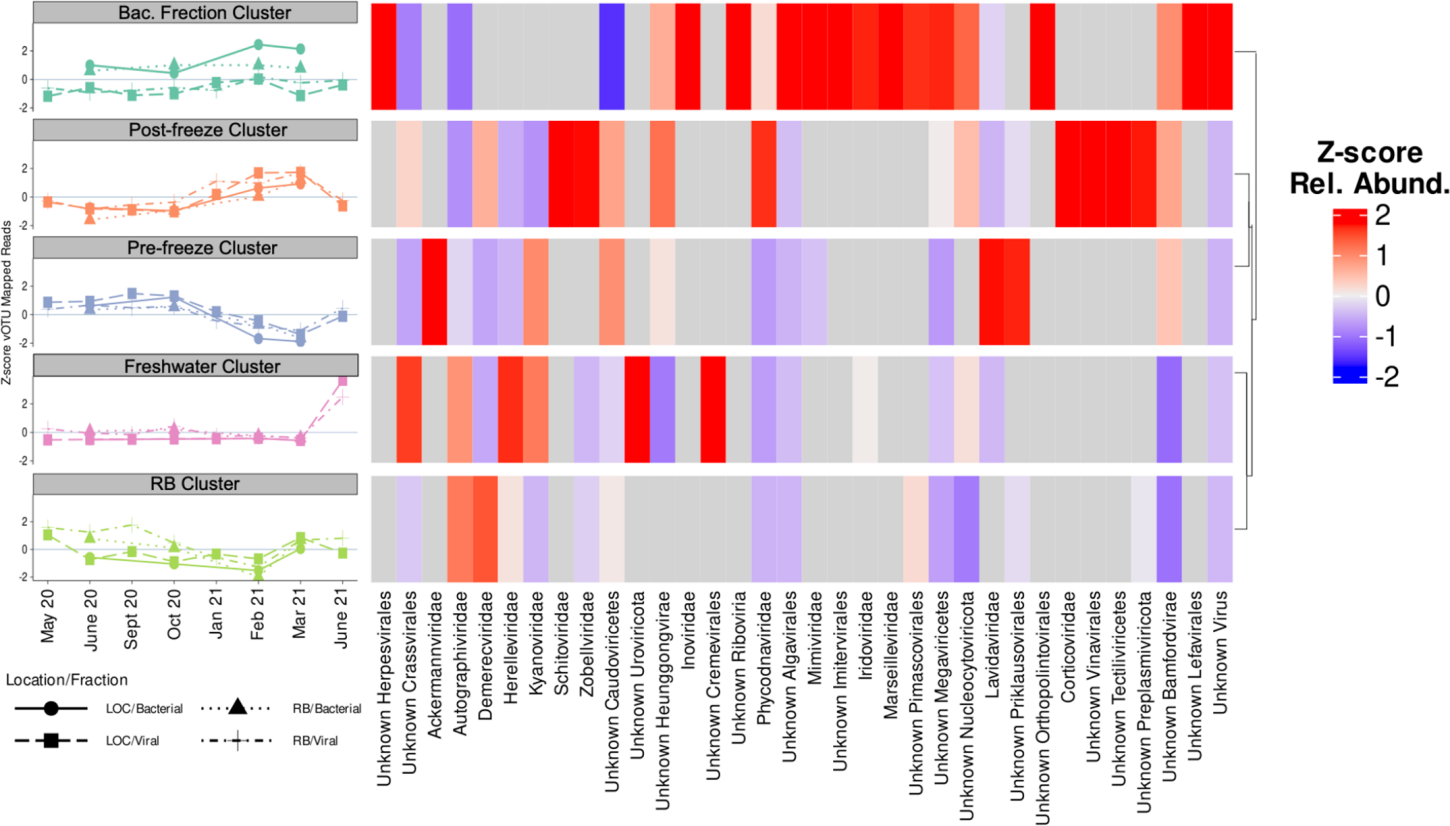
The five clusters generated by k‐mediod clustering of the vOTU relative abundances. Dynamics of representative sequences from each cluster are shown as *Z*‐scores on the left. On the right is the *Z*‐score total relative abundances across all sampling for each viral family identified. Clusters are sorted according to hierarchical clustering of the *Z*‐score transformed viral family total relative abundances. Gray bars represent viral families which were not in the associated cluster.

When clustering vOTUs according to abundance across samples, we determined the optimal number of clusters using k‐mediods and hierarchical clustering by comparing the sum of square errors within clusters of both methods using between 1–20 clusters (Figure S2). Between four and five clusters was where the steepest declines in sum of squared errors were observed, so we compared the average silhouette scores and silhouette profiles of using four and five clusters. Five clusters were optimal (Figure S2). We named the 5 k‐mediod clusters according to how prevalent representative sequences were in different samplings: (1) vOTUs abundant in the bacterial fraction cluster, (2) post‐freeze cluster, (3) pre‐freeze cluster, (4) freshwater cluster, and (5) the RB site cluster (Figure 4, Figure S3). When comparing the distribution of all vOTUs across the clusters, the vOTUs abundant in the bacterial fraction and post‐freeze clusters were most similar to each other and were most prevalent in Jan 2021 through March 2021 (Figure 4). The pre‐freeze and the RB site clusters were most prevalent in samples taken from the LOC site in May 2020 and the RB site in October 2020, respectively (Figure 4). Taxonomic assignment of the clusters showed that the bacterial cluster was enriched in families within the class Megaviricetes (Figure 4). While the post‐freeze cluster was enriched in *Zobellviridae*, *Schitoviridae*, and *Corticoviradae* and its associated higher taxonomic levels. The pre‐freeze cluster was enriched in virophages (*Lavidaviridae*) and the associated higher taxonomic levels (Figure 4). Finally, the RB site and freshwater clusters were enriched in phages particularly from the families *Autographiviridae* and *Kyanoviridae* (Figure 4).

**FIGURE 5:**
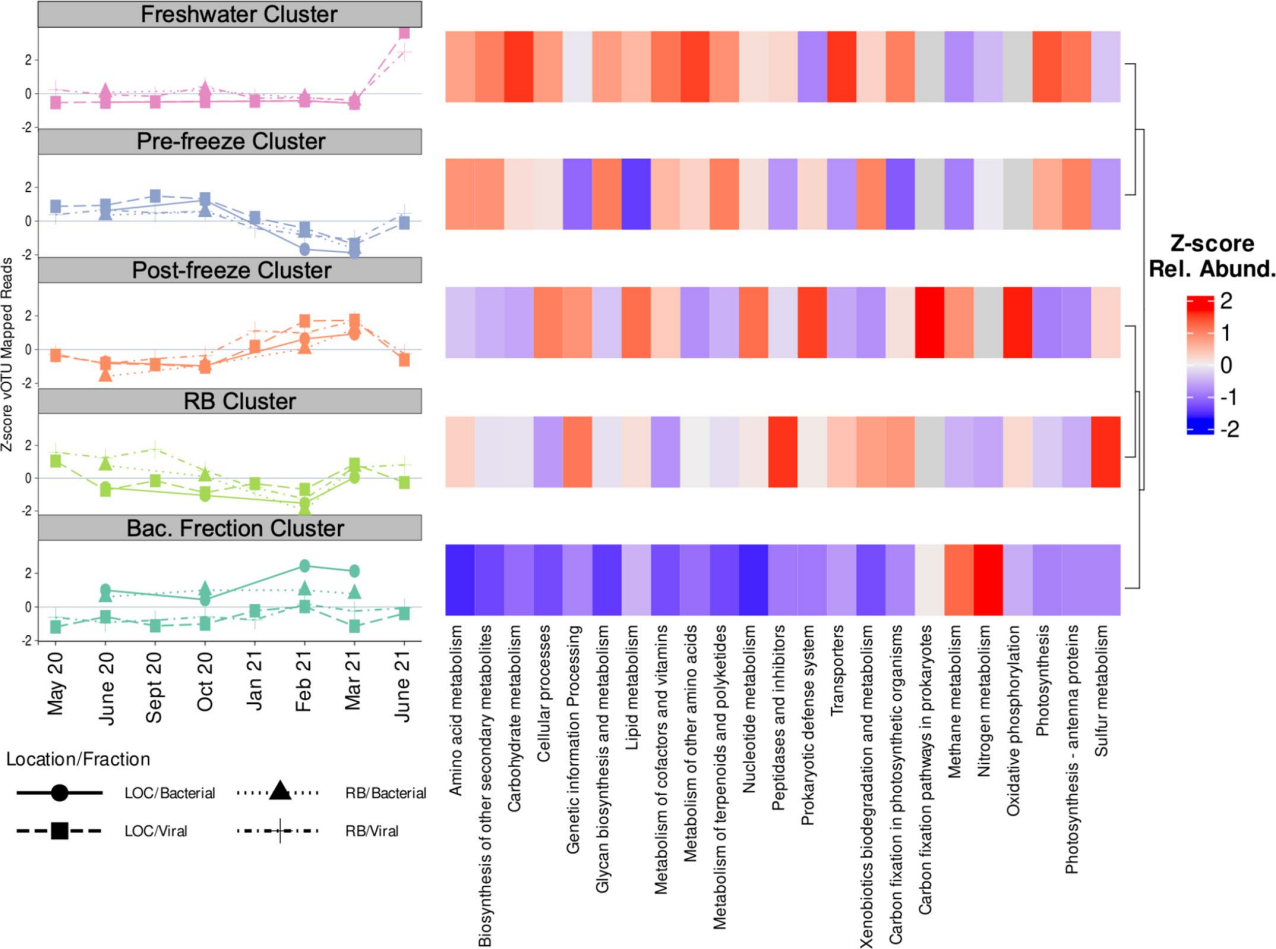
The five clusters generated by k‐medoid clustering of the vOTU relative abundances. Dynamics of representative sequences from each cluster are shown as *Z*‐scores on the left. On the right is the *Z*‐score total relative abundances across all samplings for each KEGG pathway identified in AMGs. Clusters are sorted according to hierarchical clustering of the *Z*‐score transformed total relative abundances of each KEGG pathway. Gray bars represent pathways which were not in the associated cluster.

When analyzing the relative abundances of AMGs in the vOTU clusters, we found that the freshwater and the pre‐freeze clusters were more similar (Figure 4), in contrast to the groupings based on vOTU abundances (Figure S3) and the groupings by viral family (Figure 5). The main difference between the AMGs and the other groupings is that the RB site and freshwater clusters did not group together when examining AMGs. We found that the vOTUs abundant in the bacterial fraction cluster carried relatively fewer AMGs for every metabolism except for methane and nitrogen metabolisms. The pre‐freeze and freshwater clusters were enriched in photosynthesis‐related AMGs (Figure 5). The post‐freeze and the RB site clusters were enriched in carbon fixation pathways in prokaryotes, oxidative phosphorylation, and sulfur metabolism AMGs (Figure 5).

**FIGURE 6:**
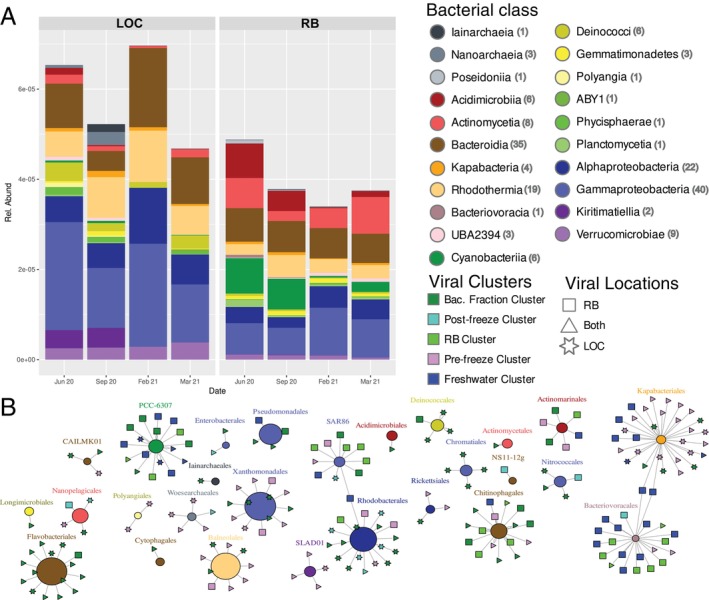
(A) Stacked bar histograms of the MAG relative abundances at the class level. (B) Network depiction of the vOTUs predicted to infect MAGs. Circles are MAGs grouped at the Order level, colored at the class level, and their sizes are relative to their total relative abundances across all samples. The number of MAGs associated with each class is identified in parentheses in the legend. vOTU shapes are based on the source of the contigs associated with the vOTUs (see Figure 2A) and colored based on the clusters they were assigned to (see Figures 4 and 5, and Figure S1).

To assess virus–host interactions, we generated 173 medium to high quality MAGs from the DNA obtained from the eight bacterial fraction metagenomes. The most common classes were Alphaproteobacteria (6.2%–17.8%), Gammaproteobacteria (8.5%–25.2%), Bacteroidia (14.1%–36.6%), and Rhodothermia (4.9%–17.2%) (Figure 6A). Cyanobacteria was one of the most abundant classes identified at the RB site, accounting for up to 18% of the community prior to the winter storm in February 2021. Other notable bacterial groups enriched at the RB site compared to the LOC site included Acidimicrobiia, Actinomycetia, Bactervoracia, Gemmatimonadetes, Planctomycetia, Poseidoniia, and UBA2394. We linked vOTUs to their potential hosts (the MAG collection) using iPHoP and found 202 vOTUs connected to 78 of the MAGs generated. Despite the relatively low abundances of Kapabacteriales, Bacteriovoracales, PCC‐6307 (Cyanobacteria), and Chitinophagales, we found some of the highest numbers of vOTUs associated with these orders (Figure 6B). We did not observe bacterial orders with connections to vOTUs from one specific location, except for Actinomarinales, for which every vOTU associated with it was sourced from the RB site (Figure 6B). Similarly, very few virus–host connections were exclusive to one viral cluster, except for the vOTUs abundant in the bacterial fraction cluster linked to Flavobacteriales, Deinococcales, and Chromatiales (Figure 6B). SLAD01 (Kiritimatiellia) was also found to only have viruses from the pre‐freeze cluster (Figure 6B). We found three viruses capable of infecting more than one order of bacteria, all assembled solely from the RB site and clustered to the freshwater cluster (Figure 6B). Two of these viruses were linked to both Kapabacteria and Bacteriovoracia, which belong to different phyla. The third virus was linked to Rhodobacterals (Gammaproteobacteria) and SAR86 (Alphaproteobacteria).

## 4 Discussion

Hypersaline environments harbor unique, diverse, and extremely dense bacterial and viral communities (Brum et al. 2005; Guixa‐Boixareu et al. 1996; Paul and Mormile 2017; Pedrós‐Alió et al. 2000; Williams 2002). These communities are sensitive to shifts in salinity, whether by increases in evaporation or dilution (Bettarel et al. 2011; Emerson et al. 2012; Motlagh et al. 2017; Roux et al. 2016; Santos et al. 2011; Uritskiy et al. 2019). A changing climate, water scarcity, and increased temperatures coupled with increasing magnitudes and frequencies of flooding events in coastal ecosystems present a need to examine how hypersaline viral communities respond to pulse disturbances (AghaKouchak et al. 2021; Coumou and Rahmstorf 2012; Donat et al. 2016; Knutson et al. 2015; Paul and Mormile 2017; Satoh et al. 2022). Here, we presented the first viral metagenomes from the Laguna Madre system, providing a baseline for the virus‐host associations, as well as a characterization of novel viruses. Our results showed how viral community composition can be altered by freshwater inundation while the functional potential of the viral community was more impacted by extreme cold weather events.

### 4.1 Viral Communities Were Unique Spatially and Temporally

Similar to previous studies, we found low connectivity between the sites investigated (Dávila‐Ramos et al. 2019; Emerson et al. 2012; Roux et al. 2016) (Figure 2A). In addition, we found that the viral assemblages were largely unique across time with 10,419 vOTUs (25% of all vOTUs) being specific to just one sample. Comparison of the taxonomic predictions of genes in the vOTUs showed that diversity, evenness, and richness were all significantly higher at the RB site compared to the LOC site. This contrasts previous viral studies of hypersaline systems that found a much higher diversity in more saline environments (Dávila‐Ramos et al. 2019; Emerson et al. 2012; Roux et al. 2016), although the highest salinity recorded in this study was lower than previous studies. The lower observed diversity in LOC compared to RB could be explained by not sampling a salinity in the range of previous studies or other confounding factors such as hydrology, host biology, or nutrient inputs having a greater impact than salinity. When assessing the origin of viral sequences by comparing with IMG/VR virus origins, those from marine, freshwater, and unknown origin made up approximately 25% of the LOC community each (Figure 2C). This likely reflects the ephemeral nature of the LOC site in which water from the Laguna Madre is the primary input in the absence of a rain event. Furthermore, the higher levels of salinity here likely sustain a unique assemblage of marine viruses when not perturbed, similar to the differences seen across salinity gradients in other hypersaline environments (Bettarel et al. 2011; Emerson et al. 2012; Roux et al. 2016). In contrast to the high level of freshwater viruses found at LOC, freshwater viruses have been shown to be distinct from those associated with marine and hypersaline environments (Logares et al. 2009; Roux et al. 2012). However, freshwater viral communities are known to be influenced by site‐specific environmental factors even when in close proximity; thus, the ephemeral nature of LOC could contribute to the unique freshwater viruses found there (Figure 2C) (Elbehery and Deng 2022; Saxton et al. 2016).

We found that the RB site contained higher relative abundances of viruses associated with algae and cyanobacteria (*Phycodnaviridae* and *Kyanoviridae)*, however, auto‐fluorescing cells were significantly higher at the LOC site. Higher levels of Kyanoviridae is consistent with the higher levels of cyanobacteria found at RB site particularly prior to the freeze events in June and September 2022 (Figure 6). These cyanobacteria were largely from the order PCC‐6307 (~11%–17% of the community) which contains the genera *Synechococcus* and *Prochlorococcus* consistent with a marine environment. Cira and Wetz (2019) have noted a higher abundance of the brown tide algae *Aureoumbra lagunensis*, known for secreting exopolymeric substances (Liu and Buskey 2000), near the LOC site compared with other sites throughout the Laguna Madre. The unique environmental (i.e., salinity or temperature) and biological (i.e., *Lavidaviridae*, exopolymeric substances) characteristics of the LOC site could be acting as controls of viruses associated with algae (*Phycodnaviridae*), reducing top‐down pressures. The combined effects of salt stress, virophages, and a thick EPS layer could be allowing hosts in the LOC site to reduce the impact of phage lysis on the community. Further research into the specific mechanisms that influence the infectivity and contact rates of viruses and their hosts in these systems would help to elucidate this observed difference in the relative abundance of algal viruses found at each site.

Investigation into the virus–host interactions revealed no apparent trends between the vOTU source location or clusters associated with a disturbance and who they infect. The most notable trend in virus–host interactions was the relatively large number of viruses associated with bacterial orders with lower total relative abundances across all sampling points. Viral‐induced mortality of low abundance bacterial species has been proposed to regulate species with competitive advantages (Bouvier and Del Giorgio 2007). Viral control of particular hosts could explain the high number of viruses and low infection rates, especially if viral survival rates are higher in hypersaline environments (Bettarel et al. 2011). Further investigation is needed to determine if viruses are more abundant in this specific system and quantitative measures, such as qPCR, over finer timescales would help elucidate specific virus–host interactions. Additionally, we used an in silico approach to connect viral sequences to hosts, which is limited by the current knowledge of virus and bacterial datasets, which could explain the large number of connections to cyanobacteria. Future studies could utilize in vitro methods of virus‐host linkage such as Hi–C or culture‐based approaches to better assess virus–host interactions within hypersaline environments.

### 4.2 Changes in Viral Communities Followed Pulse Disturbances

Clustering of the vOTUs revealed that community composition of viruses was most different in samples where salinity was low and viral AMGs were most different in the aftermath of the freeze events in January and February 2021. The vOTUs from these disturbance clusters consistently grouped apart from each other when investigating the vOTU and family level relative abundances. The freshwater cluster, or cluster enriched in sequences found in June 2021, always clustered with the RB site cluster. This indicates a similarity between the viral communities introduced by freshwater inflows and those typical of the less saline RB site. These findings are similar to what has been found in coastal systems when pulse disturbances, such as hurricanes and extreme rain events, introduce freshwater associated species that can persist for weeks (Balthis et al. 2006; Du et al. 2019; Park et al. 2007; Steichen et al. 2020; Walker et al. 2022; Williamson et al. 2014; Woods et al. 2022). Our results show how pulse disturbances can homogenize viral communities within a hypersaline system and shift them away from the unique viral communities.

The long term (13 month) experimental design allowed for the comparison of viral communities that are typical of the Laguna Madre in two different salinity regimes. Additionally, the ability to sample within days of two freeze events in January and February 2021 and a large freshwater influx in June 2021 allowed for a comparison of the communities observed throughout the year and those observed following these pulse disturbances. Despite the changes due to pulse disturbances, we observed that the functional potential of the viral communities in the freshwater cluster was more similar to the pre‐freeze cluster, as both were enriched in photosynthetic AMGs. Similarly, the post‐freeze and the RB site clusters were enriched in oxidative phosphorylation and sulfur metabolisms, despite the differences observed at the taxonomic and vOTU levels. Our results could be interpreted as viral communities being functionally redundant leading to resilient ecosystem functions (Uritskiy et al. 2019; Walker et al. 2022). The observed changes could also be driven by other factors not captured in the experiment, such as changes in host populations, changes in viral life strategy brought on by perturbation, or changes in the chemical or hydrological factors influencing the two sites. Similar freeze events where water temperatures remain above 0°C have been linked to the expansion of *A. lagunensis* blooms with nutrient release from fish kills and decreased grazing activity; confounding factors such as this could also impact viral communities (Buskey et al. 2001; Deyoe and Suttle 1994; Whitledge 1993). This pilot study provides valuable information regarding the potential for pulse disturbances to temporarily alter viral community composition and functional potential of hypersaline environments. However, it remains unclear if these changes lead to differences in how viruses are interacting with hosts; future studies should utilize metatranscriptomics, Hi–C sequencing, stable isotope probing, or biomarkers to further elucidate virus‐host interactions and the ecosystem consequences. The changes in the relative abundances of key metabolisms, such as photosynthesis and sulfur metabolisms, indicate that different pulse disturbances have different short‐term impacts on the rates of biogeochemical cycling. Efforts to quantify the changes in different key functional metabolisms following different types of pulse disturbances will help resolve the changes to biogeochemical cycling following pulse disturbances.

## 5 Conclusion

Hypersaline environments are unique ecosystems and large reservoirs of viral diversity. Previous research has shown that hypersaline viral communities are distinct across salinity gradients; however, less is known about the potential for salinity fluctuations to alter community composition and function. Anthropogenic threats, including increased severity of droughts, higher water consumption, and increased severity and frequency of extreme rain events, threaten to disturb these communities (AghaKouchak et al. 2021; Coumou and Rahmstorf 2012; Donat et al. 2016; Knutson et al. 2015; Paul and Mormile 2017; Satoh et al. 2022). By investigating two connected viral communities within a hypersaline coastal estuary for 13 months, we found two distinct viral communities. We investigated the potential for functional redundancy to maintain ecosystem function through the disturbance and presumably the recovery process (Uritskiy et al. 2019; Walker et al. 2022). We demonstrated that different types of pulse disturbances lead to alterations in the relative abundances of key metabolic processes present in viral communities. The patterns observed here provide hypotheses regarding environmental controls on viral community composition that can be tested in higher resolution temporal studies. Future studies should aim to quantify the effects of pulse disturbances on both viral and overall community function either through direct measurements of activity or transcription of genes. Additionally, finer scale sampling would allow for a clearer view of the post‐disturbance community changes and recovery process. Our work presents the potential for diverse microbial communities to respond to extreme ecosystem fluxes and highlights the need to better quantify alterations to important biogeochemical cycles.

## Author Contributions

**Jordan R. Walker:** investigation, writing – original draft, methodology, visualization, writing – review and editing, software, formal analysis, data curation. **Paxton T. Bachand:** investigation, methodology. **Jeffrey W. Turner:** conceptualization, funding acquisition, writing – review and editing, project administration, supervision, resources. **Jessica M. Labonté:** conceptualization, funding acquisition, writing – review and editing, project administration, supervision, resources.

## Funding

This work was supported by the Texas General Land Office Coastal Management Program (NA19NOS4190106).

## Conflicts of Interest

The authors declare no conflicts of interest.

## Supporting information


**Figure S1:** Comparisons of diversity indices between sites.

**Figure S2:** Summary statistics of k‐mediod cluster comparisons

**Figure S3:** Heatmap of all vOTUs across all sampling.

**Table S1:** Metagenomic sequencing, quality control, and assembly statistics.

## Data Availability

The data that support the findings of this study are openly available in NCBI Sequence Read Archive at https://www.ncbi.nlm.nih.gov/bioproject/PRJNA1168473/, reference number PRJNA1168473.
