# Supplementary material for: Viral Assemblages of a Hypersaline Estuary Show Divergent Responses to Freshwater and Temperature Disturbances
Source: Environ Microbiol Rep. 2026 May 8;18(3):e70354. doi: 10.1111/1758-2229.70354 (PMC13154383; doi:10.1111/1758-2229.70354)
Supplement: Supplementary file 1 — Figure S1: Comparisons of diversity indices between sites. [file EMI4-18-e70354-s003.docx]

7.5

0.998


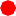

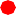

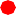

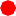

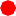

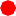

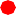

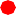

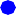

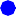

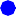

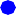

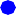

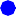

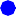

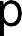


Shannon Index

Wilcoxon

= 0.0015

6000

0.90


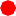

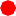

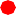

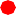

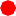

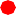

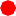

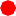

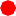

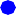

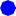

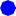

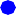

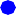

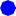

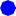

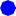

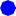

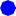

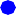

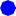

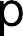


Chao1 Index

Wilcoxon

= 0.00098


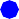

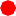

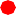

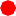

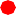

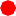

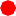

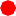

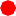

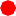

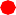

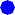

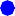

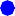

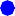

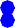

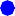

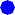

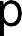


Pielou Index

Wilcoxon

= 0.027

7.0

6.5

0.996

4000

0.88

0.86

0.994

2000

0.84

6.0


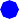

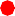

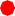

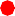

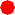

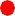

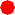

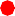

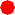

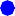

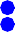

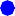

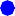

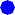

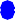

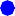

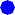

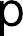


Simpson Index

Wilcoxon

= 0.012

0.82

0.992

LOG RB LOG RB LOG RB LOG RB

Supplementary Figure 1: Boxplots of the vOTU diversity (Shannon and Simpson), richness (Chao1), and evenness (Pielou) at the LOC (red) and RB sites (blue).
