# Supplementary material for: Viral Assemblages of a Hypersaline Estuary Show Divergent Responses to Freshwater and Temperature Disturbances
Source: Environ Microbiol Rep. 2026 May 8;18(3):e70354. doi: 10.1111/1758-2229.70354 (PMC13154383; doi:10.1111/1758-2229.70354)
Supplement: Supplementary file 2 — Figure S2: Summary statistics of k‐mediod cluster comparisons [file EMI4-18-e70354-s001.docx]

**A**

350000

Within Cluster Sum Square Error

300000

250000

200000

Method


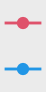
Hierarchical K−Medoids

**B**

1.00

0.75

Silhouette width Si

0.50

0.25

0.00

Clusters silhouette plot Average silhouette width: 0.16

cluster


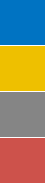
1

2

3

4

**C**

1.00

0.75

Silhouette width Si

0.50

0.25

0.00

Clusters silhouette plot Average silhouette width: 0.18

cluster


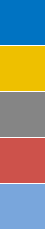
1

2

3

4

5


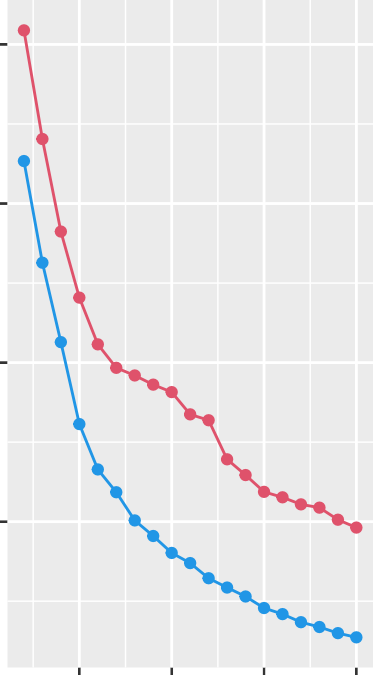

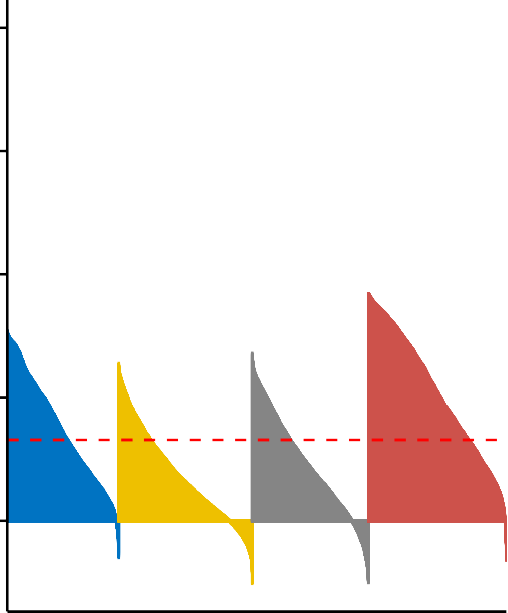

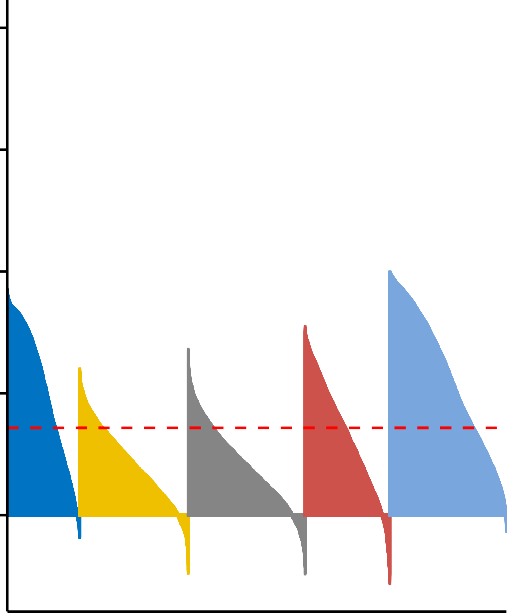
5 10 15 20

Number Clusters

Supplementary Figure 2: (A) Sum of square errors of clustering utilizing a hierarchical (red) and k-medoids (blue) approach for 1 through 20 clusters. (B-C) Silhoutte profiles of k-medoids clustering using 4 (B) and 5 (C) clusters.
