# Supplementary material for: Viral Assemblages of a Hypersaline Estuary Show Divergent Responses to Freshwater and Temperature Disturbances
Source: Environ Microbiol Rep. 2026 May 8;18(3):e70354. doi: 10.1111/1758-2229.70354 (PMC13154383; doi:10.1111/1758-2229.70354)
Supplement: Supplementary file 3 — Figure S3: Heatmap of all vOTUs across all sampling. [file EMI4-18-e70354-s004.docx]

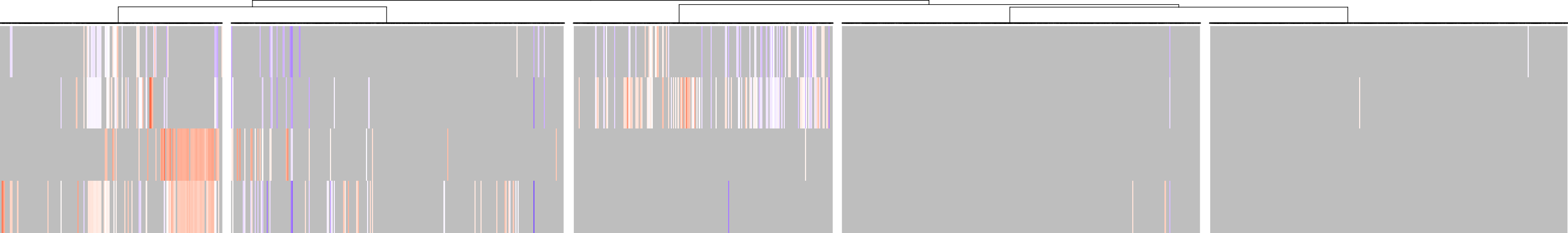

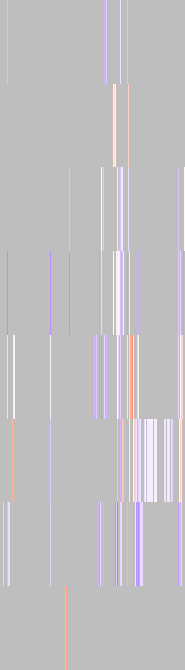

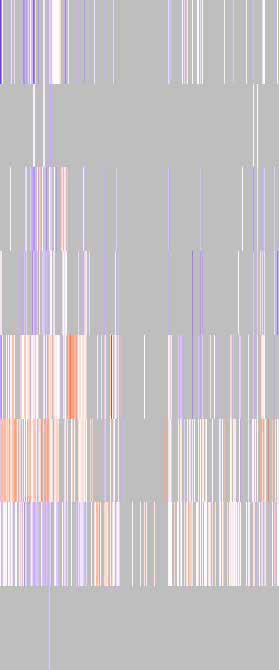

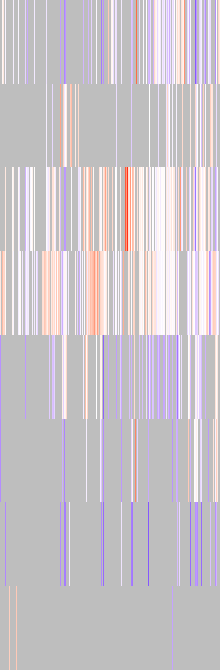

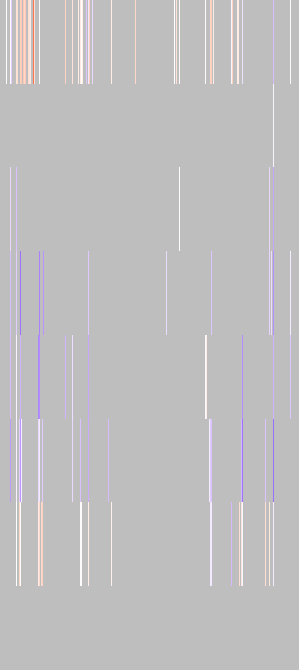

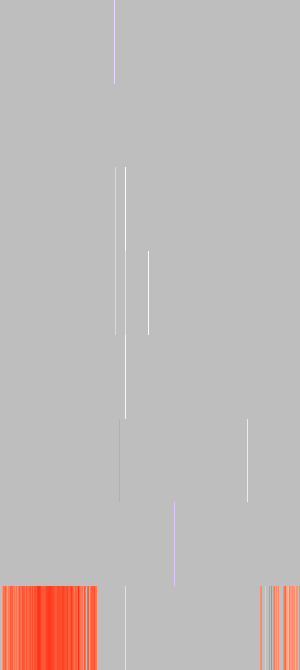

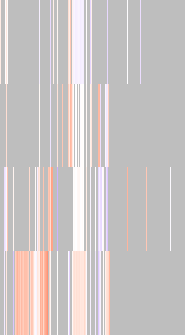

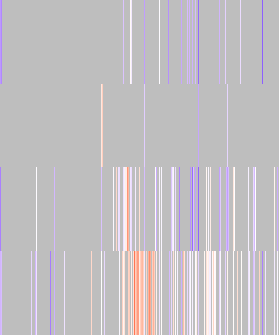

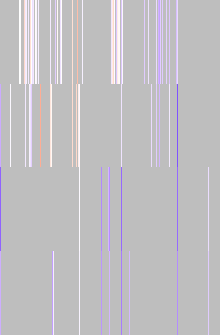

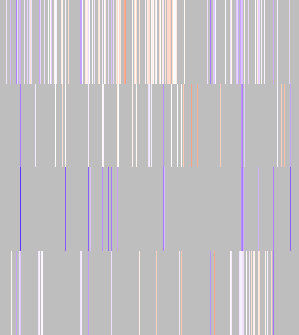

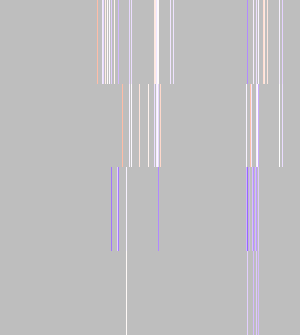

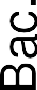

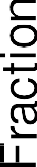

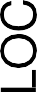

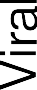

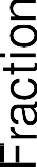

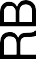

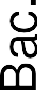

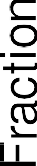

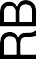

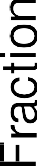

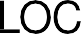

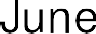

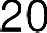

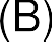

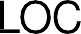

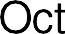

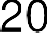

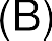

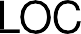

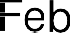

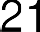

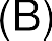

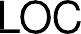

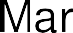

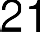

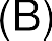

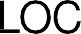

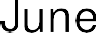

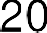

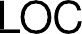

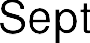

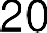

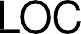

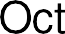

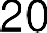

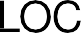

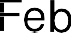

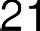

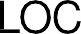

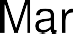

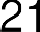

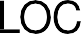

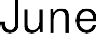

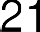

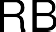

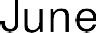

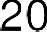

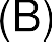

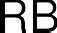

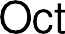

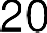

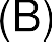

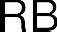

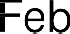

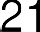

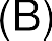

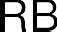

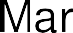

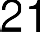

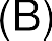

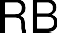

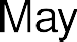

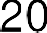

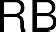

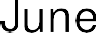

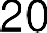

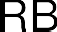

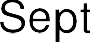

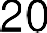

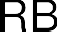

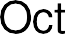

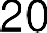

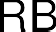

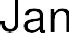

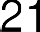

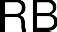

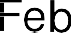

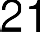

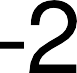

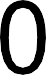

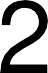

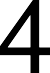

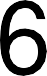

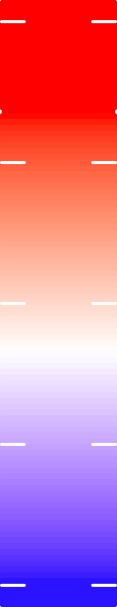

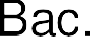

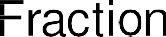

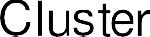

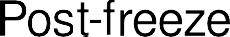

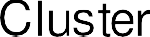


# Variance stabilized, detrended, scaled relative abundance

Supplementary Figure 3: Heat map of the variance stabilized, detrended, and scaled relative abundances of the vOTUs. The vOTU (columns) clustering was performed according to the k-medoids algorithm utilized in Figure 4, while the clustering of the samples (rows) was done utilizing hierarchical clustering with Euclidean distances.

Gray bars represent vOTUS absent from the sample. r
