# Supplementary material for: Viral Assemblages of a Hypersaline Estuary Show Divergent Responses to Freshwater and Temperature Disturbances
Source: Environ Microbiol Rep. 2026 May 8;18(3):e70354. doi: 10.1111/1758-2229.70354 (PMC13154383; doi:10.1111/1758-2229.70354)
Supplement: Supplementary file 4 — Table S1: Metagenomic sequencing, quality control, and assembly statistics. [file EMI4-18-e70354-s002.docx]

Supplemental Table 1: Quality control and assembly statistics for metagenomes generated. The annotation M refers to merged reads and UM to reads that were not merged. Trimming is reported in the bases and reads remaining after trimming. Contamination removal and entropy masking are reported in the percentage removed or masked.

| **Sample** | **Site** | **Size** | **Merging** | | | **Artifact Trimming** | | **Adapter Trimming** | | **Common Contaminants** | | **Entropy** | **Assembly** | | |
| --- | --- | --- | --- | --- | --- | --- | --- | --- | --- | --- | --- | --- | --- | --- | --- |
|  |  |  | **Reads** | **Joined** | **M / UM** | **Remain Reads** | **Remain Bases** | **Remain Reads** | **Remain Bases** | **Reads Match** | **Bases Match** | **Masked Bases** | **Contigs** | **Bases** | **N50** |
| May 20 L1 | LOC | Vir | 41,538,088 | 8,990,729 | M | 100% | 99% | 100% | 100% | 0.00% | 0.00% | 0.00% | 571,114 | 314,983,804 | 601 |
|  |  |  |  |  | UM | 100% | 100% | 96% | 90% | 0.34% | 0.05% | 0.13% |  |  |  |
| May 20 L2 | LOC | Vir | 42,148,378 | 9,129,152 | M | 100% | 99% | 100% | 100% | 0.00% | 0.00% | 0.00% |  |  |  |
|  |  |  |  |  | UM | 100% | 100% | 97% | 92% | 0.21% | 0.03% | 0.13% |  |  |  |
| June 20 L1 | LOC | Vir | 5,289,984 | 722,325 | M | 100% | 99% | 100% | 100% | 1.18% | 0.88% | 0.00% | 116,722 | 69,844,317 | 643 |
|  |  |  |  |  | UM | 100% | 100% | 86% | 82% | 1.40% | 1.13% | 0.13% |  |  |  |
| June 20 L2 | LOC | Vir | 5,377,340 | 733,946 | M | 100% | 99% | 100% | 100% | 1.14% | 0.86% | 0.00% |  |  |  |
|  |  |  |  |  | UM | 100% | 100% | 87% | 84% | 1.32% | 1.13% | 0.13% |  |  |  |
| Sept 20 L1 | LOC | Vir | 37,534,446 | 7,132,976 | M | 100% | 99% | 100% | 100% | 0.00% | 0.00% | 0.00% | 582,953 | 322,447,287 | 611 |
|  |  |  |  |  | UM | 100% | 100% | 98% | 92% | 0.31% | 0.04% | 0.12% |  |  |  |
| Sept 20 L2 | LOC | Vir | 38,145,524 | 7,251,729 | M | 100% | 99% | 100% | 100% | 0.00% | 0.00% | 0.00% |  |  |  |
|  |  |  |  |  | UM | 100% | 100% | 98% | 94% | 0.19% | 0.03% | 0.12% |  |  |  |
| Oct 20 L1 | LOC | Vir | 45,410,538 | 7,177,436 | M | 100% | 99% | 100% | 100% | 0.00% | 0.00% | 0.00% | 777,806 | 418,112,689 | 587 |
|  |  |  |  |  | UM | 100% | 100% | 98% | 92% | 0.32% | 0.05% | 0.07% |  |  |  |
| Oct 20 L2 | LOC | Vir | 46,371,076 | 7,315,875 | M | 100% | 99% | 100% | 100% | 0.00% | 0.00% | 0.00% |  |  |  |
|  |  |  |  |  | UM | 100% | 100% | 98% | 94% | 0.20% | 0.03% | 0.07% |  |  |  |
| Jan 21 L1 | LOC | Vir | 38,174,224 | 6,268,382 | M | 100% | 99% | 100% | 99% | 0.01% | 0.01% | 0.00% | 729,929 | 432,439,795 | 652 |
|  |  |  |  |  | UM | 100% | 100% | 99% | 93% | 0.37% | 0.07% | 0.09% |  |  |  |
| Jan 21 L2 | LOC | Vir | 39,000,980 | 6,392,236 | M | 100% | 99% | 100% | 99% | 0.01% | 0.01% | 0.00% |  |  |  |
|  |  |  |  |  | UM | 100% | 100% | 99% | 94% | 0.26% | 0.05% | 0.08% |  |  |  |
| Feb 21 L1 | LOC | Vir | 38,984,760 | 6,690,360 | M | 100% | 99% | 100% | 99% | 0.00% | 0.00% | 0.00% | 628,555 | 384,071,168 | 669 |
|  |  |  |  |  | UM | 100% | 100% | 99% | 93% | 0.31% | 0.05% | 0.09% |  |  |  |
| Feb 21 L2 | LOC | Vir | 39,877,812 | 6,832,183 | M | 100% | 99% | 100% | 100% | 0.01% | 0.00% | 0.00% |  |  |  |
|  |  |  |  |  | UM | 100% | 100% | 99% | 95% | 0.20% | 0.03% | 0.08% |  |  |  |
| March 21 L1 | LOC | Vir | 29,605,334 | 3,713,186 | M | 100% | 99% | 100% | 99% | 0.25% | 0.15% | 0.00% | 505,725 | 302,371,144 | 671 |
|  |  |  |  |  | UM | 100% | 100% | 98% | 91% | 0.54% | 0.23% | 0.08% |  |  |  |
| March 21 L2 | LOC | Vir | 30,378,946 | 3,779,124 | M | 100% | 99% | 100% | 100% | 0.23% | 0.14% | 0.00% |  |  |  |
|  |  |  |  |  | UM | 100% | 100% | 98% | 93% | 0.41% | 0.20% | 0.07% |  |  |  |
| June 21 L1 | LOC | Vir | 37,583,594 | 9,825,785 | M | 100% | 99% | 100% | 99% | 0.00% | 0.00% | 0.00% | 234,780 | 208,020,391 | 1,262 |
|  |  |  |  |  | UM | 100% | 100% | 99% | 92% | 0.39% | 0.05% | 0.20% |  |  |  |
| June 21 L2 | LOC | Vir | 38,077,868 | 10,004,323 | M | 100% | 99% | 100% | 100% | 0.00% | 0.00% | 0.00% |  |  |  |
|  |  |  |  |  | UM | 100% | 100% | 99% | 94% | 0.23% | 0.03% | 0.20% |  |  |  |
| May 20 L1 | RB | Vir | 42,843,070 | 7,282,530 | M | 100% | 99% | 100% | 99% | 0.00% | 0.00% | 0.01% | 785,683 | 496,616,648 | 736 |
|  |  |  |  |  | UM | 100% | 100% | 99% | 92% | 0.39% | 0.05% | 0.13% |  |  |  |
| May 20 L2 | RB | Vir | 43,844,104 | 7,443,345 | M | 100% | 99% | 100% | 100% | 0.00% | 0.00% | 0.01% |  |  |  |
|  |  |  |  |  | UM | 100% | 100% | 99% | 94% | 0.25% | 0.03% | 0.12% |  |  |  |
| June 20 L1 | RB | Vir | 54,441,574 | 13,026,008 | M | 100% | 99% | 100% | 99% | 0.00% | 0.00% | 0.00% | 808,265 | 528,088,541 | 768 |
|  |  |  |  |  | UM | 100% | 100% | 99% | 91% | 0.46% | 0.06% | 0.16% |  |  |  |
| June 20 L2 | RB | Vir | 54,937,822 | 13,240,489 | M | 100% | 99% | 100% | 99% | 0.00% | 0.00% | 0.00% |  |  |  |
|  |  |  |  |  | UM | 100% | 100% | 99% | 93% | 0.28% | 0.04% | 0.16% |  |  |  |
| Sept 20 L1 | RB | Vir | 49,830,480 | 12,434,443 | M | 100% | 99% | 100% | 99% | 0.00% | 0.00% | 0.00% | 888,439 | 578,815,624 | 748 |
|  |  |  |  |  | UM | 100% | 100% | 99% | 91% | 0.45% | 0.06% | 0.21% |  |  |  |
| Sept 20 L2 | RB | Vir | 50,245,248 | 12,627,626 | M | 100% | 99% | 100% | 99% | 0.00% | 0.00% | 0.00% |  |  |  |
|  |  |  |  |  | UM | 100% | 100% | 99% | 93% | 0.27% | 0.04% | 0.20% |  |  |  |
| Oct 20 L1 | RB | Vir | 47,600,518 | 12,112,876 | M | 100% | 99% | 100% | 100% | 0.01% | 0.01% | 0.00% | 866,096 | 601,843,575 | 808 |
|  |  |  |  |  | UM | 100% | 100% | 99% | 92% | 0.45% | 0.07% | 0.31% |  |  |  |
| Oct 20 L2 | RB | Vir | 47,901,998 | 12,274,251 | M | 100% | 99% | 100% | 100% | 0.01% | 0.01% | 0.00% |  |  |  |
|  |  |  |  |  | UM | 100% | 100% | 99% | 94% | 0.28% | 0.04% | 0.31% |  |  |  |
| Jan 21 L1 | RB | Vir | 39,770,958 | 8,294,793 | M | 100% | 99% | 100% | 99% | 0.00% | 0.00% | 0.00% | 739,514 | 456,741,260 | 707 |
|  |  |  |  |  | UM | 100% | 100% | 98% | 91% | 0.43% | 0.06% | 0.19% |  |  |  |
|  |  |  |  |  |  |  |  |  |  |  |  |  |  |  |  |
| Jan 21 L2 | RB | Vir | 40,184,854 | 8,440,557 | M | 100% | 99% | 100% | 99% | 0.00% | 0.00% | 0.00% |  |  |  |
|  |  |  |  |  | UM | 100% | 100% | 98% | 93% | 0.25% | 0.03% | 0.19% |  |  |  |
| Feb 21 L1 | RB | Vir | 45,155,564 | 13,703,948 | M | 100% | 99% | 100% | 100% | 0.01% | 0.01% | 0.01% | 755,965 | 500,017,108 | 720 |
|  |  |  |  |  | UM | 100% | 100% | 95% | 86% | 0.69% | 0.11% | 0.51% |  |  |  |
| Feb 21 L2 | RB | Vir | 45,018,064 | 13,786,632 | M | 100% | 99% | 100% | 100% | 0.01% | 0.01% | 0.01% |  |  |  |
|  |  |  |  |  | UM | 100% | 100% | 96% | 88% | 0.50% | 0.09% | 0.50% |  |  |  |
| March 21 L1 | RB | Vir | 39,632,896 | 7,961,732 | M | 100% | 99% | 100% | 100% | 0.01% | 0.01% | 0.00% | 724,887 | 467,604,024 | 730 |
|  |  |  |  |  | UM | 100% | 100% | 98% | 91% | 0.43% | 0.07% | 0.19% |  |  |  |
| March 21 L2 | RB | Vir | 40,236,484 | 8,067,802 | M | 100% | 99% | 100% | 100% | 0.01% | 0.00% | 0.00% |  |  |  |
|  |  |  |  |  | UM | 100% | 100% | 98% | 93% | 0.29% | 0.05% | 0.19% |  |  |  |
| June 21 L1 | RB | Vir | 38,041,482 | 8,207,192 | M | 100% | 99% | 100% | 99% | 0.03% | 0.02% | 0.00% | 486,211 | 355,015,789 | 847 |
|  |  |  |  |  | UM | 100% | 100% | 99% | 91% | 0.53% | 0.13% | 0.23% |  |  |  |
| June 21 L2 | RB | Vir | 38,624,514 | 8,359,994 | M | 100% | 99% | 100% | 99% | 0.03% | 0.02% | 0.00% |  |  |  |
|  |  |  |  |  | UM | 100% | 100% | 99% | 93% | 0.38% | 0.11% | 0.23% |  |  |  |
| June 20 L1 | LOC | Bac | 47,995,486 | 10,867,417 | M | 100% | 99% | 100% | 100% | 0.00% | 0.00% | 0.00% | 1,036,874 | 743,544,469 | 812 |
|  |  |  |  |  | UM | 100% | 100% | 98% | 92% | 0.30% | 0.04% | 0.19% |  |  |  |
| June 20 L2 | LOC | Bac | 48,941,042 | 11,098,786 | M | 100% | 99% | 100% | 100% | 0.00% | 0.00% | 0.00% |  |  |  |
|  |  |  |  |  | UM | 100% | 100% | 98% | 94% | 0.22% | 0.03% | 0.18% |  |  |  |
| Sept 20 L1 | LOC | Bac | 43,161,480 | 9,960,910 | M | 100% | 99% | 100% | 100% | 0.00% | 0.00% | 0.00% | 1,038,864 | 699,851,964 | 746 |
|  |  |  |  |  | UM | 100% | 100% | 98% | 91% | 0.36% | 0.05% | 0.27% |  |  |  |
| Sept 20 L2 | LOC | Bac | 43,758,448 | 10,133,931 | M | 100% | 99% | 100% | 100% | 0.00% | 0.00% | 0.00% |  |  |  |
|  |  |  |  |  | UM | 100% | 100% | 98% | 93% | 0.25% | 0.03% | 0.26% |  |  |  |
| Feb 21 L1 | LOC | Bac | 54,510,090 | 14,783,082 | M | 100% | 99% | 100% | 100% | 0.00% | 0.00% | 0.02% | 604,157 | 529,881,343 | 1,221 |
|  |  |  |  |  | UM | 100% | 100% | 99% | 91% | 0.66% | 0.11% | 0.63% |  |  |  |
| Feb 21 L2 | LOC | Bac | 55,379,656 | 15,022,492 | M | 100% | 99% | 100% | 100% | 0.00% | 0.00% | 0.02% |  |  |  |
|  |  |  |  |  | UM | 100% | 100% | 99% | 93% | 0.56% | 0.10% | 0.62% |  |  |  |
|  |  |  |  |  |  |  |  |  |  |  |  |  |  |  |  |
| March 21 L1 | LOC | Bac | 49,108,650 | 12,032,256 | M | 100% | 99% | 100% | 100% | 0.00% | 0.00% | 0.02% | 857,561 | 689,430,756 | 983 |
|  |  |  |  |  | UM | 100% | 100% | 99% | 91% | 0.63% | 0.10% | 0.44% |  |  |  |
| March 21 L2 | LOC | Bac | 49,760,760 | 12,196,246 | M | 100% | 99% | 100% | 100% | 0.00% | 0.00% | 0.02% |  |  |  |
|  |  |  |  |  | UM | 100% | 100% | 99% | 93% | 0.53% | 0.09% | 0.42% |  |  |  |
| June 20 L1 | RB | Bac | 38,692,766 | 5,459,816 | M | 100% | 99% | 100% | 100% | 0.00% | 0.00% | 0.01% | 988,807 | 701,682,733 | 800 |
|  |  |  |  |  | UM | 100% | 100% | 99% | 93% | 0.33% | 0.04% | 0.25% |  |  |  |
| June 20 L2 | RB | Bac | 39,589,462 | 5,551,972 | M | 100% | 99% | 100% | 100% | 0.00% | 0.00% | 0.01% |  |  |  |
|  |  |  |  |  | UM | 100% | 100% | 99% | 95% | 0.23% | 0.03% | 0.24% |  |  |  |
| Sept 20 L1 | RB | Bac | 43,362,214 | 6,610,222 | M | 100% | 99% | 100% | 100% | 0.01% | 0.00% | 0.04% | 1,029,378 | 675,467,381 | 689 |
|  |  |  |  |  | UM | 100% | 100% | 99% | 92% | 0.54% | 0.10% | 0.65% |  |  |  |
| Sept 20 L2 | RB | Bac | 44,180,250 | 6,714,063 | M | 100% | 99% | 100% | 100% | 0.01% | 0.00% | 0.04% |  |  |  |
|  |  |  |  |  | UM | 100% | 100% | 99% | 94% | 0.43% | 0.08% | 0.64% |  |  |  |
| Feb 21 L1 | RB | Bac | 48,123,588 | 10,101,037 | M | 100% | 99% | 100% | 100% | 0.00% | 0.00% | 0.08% | 1,165,460 | 767,429,213 | 689 |
|  |  |  |  |  | UM | 100% | 100% | 98% | 90% | 0.80% | 0.15% | 1.37% |  |  |  |
| Feb 21 L2 | RB | Bac | 48,524,952 | 10,177,243 | M | 100% | 99% | 100% | 100% | 0.00% | 0.00% | 0.08% |  |  |  |
|  |  |  |  |  | UM | 100% | 100% | 99% | 92% | 0.67% | 0.13% | 1.35% |  |  |  |
| March 21 L1 | RB | Bac | 39,473,220 | 7,811,148 | M | 100% | 99% | 100% | 100% | 0.00% | 0.00% | 0.03% | 836,797 | 638,418,255 | 881 |
|  |  |  |  |  | UM | 100% | 100% | 99% | 92% | 0.47% | 0.07% | 0.44% |  |  |  |
| March 21 L2 | RB | Bac | 40,002,636 | 7,952,264 | M | 100% | 99% | 100% | 100% | 0.00% | 0.00% | 0.02% |  |  |  |
|  |  |  |  |  | UM | 100% | 100% | 99% | 94% | 0.32% | 0.05% | 0.43% |  |  |  |
